# Rett Syndrome in Males: A Case Report and Review of Literature

**DOI:** 10.7759/cureus.3414

**Published:** 2018-10-04

**Authors:** Gurneet Chahil, Anudeep Yelam, Pradeep C Bollu

**Affiliations:** 1 Neurology, University of Missouri, Columbia, USA

**Keywords:** rett syndrome

## Abstract

Rett syndrome (RTT) is a neurodevelopmental disorder in which a period of normal development is followed by regression of previously acquired skills. RTT was originally thought to be present exclusively in females. However, advances in genetic testing and phenotypic identification revealed that it is not a female-only disorder as cases of males with similar phenotype were reported. RTT was considered lethal in males as it has an X-linked dominant inheritance. The purpose of this review is to report a case of RTT in young male and elaborate genetics and phenomenology of this disorder in males.

## Introduction

Rett syndrome (RTT) is a neurodevelopmental disorder that is primarily seen in females [1]. It was first described by Andreas Rett in 1966 in two young girls noting normal development in the first year of life followed by regression with loss of previously developed skills [2]. It is characterized by a brief period of normal development followed by loss of acquired skills like hand use and speech, autistic features, gait abnormalities, head growth deceleration, stereotypic hand movements, and breathing irregularities [3]. Majority of the patients with RTT are females [4].

## Case presentation

A 16-year-old male patient was brought by his parents to a genetics clinic with complaints of developmental delay and tremor. He was a full-term infant with an uneventful delivery. He started walking around 18 months of age, spoke his first words at the age of two. Other problems included nervousness, immature behaviors, lack of eye contact during conversations, and aggressive behavior. His mother reported that he began to have a tremor in the hands around three years of age. Diagnostic workup included magnetic resonance imaging (MRI) of the brain, urine organic and amino acids, lactate, pyruvate, and lead levels along with chromosomal and DNA analysis for fragile X which were all unremarkable. His family history was significant for mental retardation. Maternal grandmother had three mentally retarded brothers with tremors, two of whom died in their forties. The patient’s brother also seems to have a speech delay along with tremors since the age of three.

His tremors worsened gradually, and he started to have difficulties with fine motor control including difficulty with drinking out of a cup along with increased aggression and behavioral changes. His teachers reported that he was biting, kicking, spitting and getting into conflicts with other children. He was seen by a child psychiatrist at that time and was started on risperidone, valproic acid and Adderall (amphetamine and dextroamphetamine) which seemed to help with his behaviors.

On examination, he has high nasal bridge, slightly down-slanting palpebral fissures, long philtrum, and thin upper lip. On neurological exam, he has slightly increased deep tendon reflexes throughout. Babinski sign was positive on the right, but a normal plantar response was noted on the left side. Bilateral hand tremors were noted, both at rest and in action. He was walking slowly without much arm swing and had a slightly stooped forward posture. A full psychological evaluation was done which showed a Leiter scale IQ of 91. The Vineland adaptive behavior scale showed functioning at the 19-month level. On the childhood autism rating scale (CARS) he scored 31 to 32, consistent with mild autism. He was enrolled in a special education program and speech therapy.

Genetic testing was ordered in both the patient and his brother that was positive for a Rett syndrome methyl-CpG-binding protein 2 (MECP2) mutation, A140V in both the boys.

## Discussion

Research and literature have shown a strong correlation between mutations in the methyl-CpG-binding protein 2 (MECP2) and RTT [5]. Originally, the near complete absence of males with classic RTT postulated a lethal effect of the MECP2 mutation in males [6, 7]. Contrary to this belief, MECP2 mutations have been reported and documented in male patients that displayed a wide assortment of presentations including but not limited to severe neurodevelopmental disabilities, congenital encephalopathy, and classical RTT [8]. Those who are suspected of having RTT or being affected by mutations in MECP2 gene can be further categorized into several groups [9]: 1) sporadic cases of males who meet the inclusion criteria for RTT, 2) males with Klinefelter's syndrome, 3) males who are mosaic for mutation of MECP2 gene, 4) males with severe neonatal encephalopathy in families with RTT. The MeCP2 protein is mapped to Xq28 and encodes methyl-CpG-binding protein 2 (MeCP2) [10] which is more abundant in brain. This could be the reason why the brain is more sensitive to abnormal MeCP2 than other tissues in the body.

It is unclear how MECP2 mutation causes RTT. MECP2 has two functional domains, a methyl CpG binding domain, and a transcriptional repression domain [11]. Mutations associated with RTT are mainly localized to these two functional domains. MECP2 can modulate, repress or activate transcription and can also promote genomic imprinting. So, MeCP2 deficiency or loss of function results in aberrant gene expression leading to RTT [12]. Other theories postulate that MeCP2 deficiency leads to failure in the maturation and maintenance of synapses in multiple brain systems [13]. It can also alter brain cholesterol metabolism thus disrupting neuronal development [14].

It is to be noted that MECP2 gene mutations are extremely common in classic cases of RTT and can be seen in up to 95% of cases [15]. The types of genetic mutations most often observed in the MECP2 gene are missense, nonsense, and frameshift mutations. Depending on the type and region of mutation, the severity of the phenotype can greatly vary. For instance, a mutation in the nuclear localization signal (NLS) region of the MECP2 can present with a more severe phenotype whereas a C-terminal deletion of the MECP2 gene would present with a milder phenotype. The R133C mutation is generally associated with a milder variant of RTT often with preserved speech [8].

The same MECP2 mutations that cause classic RTT in females can cause cases of neonatal encephalopathy and death in males with a normal karyotype in the first year of life. At the same time, MECP2 mutations that do not manifest RTT in females can lead to moderate or profound mental retardation in males. An interesting fact is that both a loss of function or gain in MECP2 can manifest similar clinical presentations. There have been cases of boys reported to have duplications of Xq28 located on the MECP2 locus and have displayed variants of RTT phenotypes. Studies have also shown that in male mice, the overexpression of Mecp2 causes a progressive neurological disorder [16].

The diagnosis of RTT is based on history, examination, clinical criteria and genetic testing [3]. In our patient, history of initial normal development followed by regression and clinical characteristics along with positive MECP2, A140V mutation which substitutes amino acid alanine to valine at residue 140 in the methyl binding domain of the MECP2 gene aided in the diagnosis of RTT.

There is no specific therapy for RTT. Management consists of multidisciplinary approach along with treatment of associated conditions, providing physical, occupational, speech therapy. Animal studies have proven that re-expression of Mecp2 in symptomatic Mecp2 mice markedly improved function and longevity thus suggesting therapeutic targets in humans [14].

## Conclusions

Rett syndrome is a neurodevelopmental disorder that is commonly seen in girls. Although rare, physicians should not dismiss the diagnosis of Rett syndrome in males. It is imperative to do a genetic evaluation of males presenting with Rett-like symptoms and to be aware of the diverse phenotypic variation in RTT. Management is supportive, treating any associated conditions along with physical, occupational, speech therapy for their daily functioning. There is still limited literature on RTT in males, and this needs to be explored more.
